# Current Perspectives on Atrial Amyloidosis: A Narrative Review

**DOI:** 10.31083/j.rcm2502073

**Published:** 2024-02-20

**Authors:** Marco Tana, Claudio Tana, Maria Domenica Guglielmi, Arianna Stefanelli, Cesare Mantini, Ettore Porreca

**Affiliations:** ^1^Internal Medicine and Cardiovascular Ultrasound Unit, Medical Department, St Annunziata Hospital, 66100 Chieti, Italy; ^2^Department of Innovative Technologies in Medicine and Dentistry, G. D’Annunzio University of Chieti-Pescara, 66100 Chieti, Italy; ^3^Geriatrics Clinic, St Annunziata Hospital, 66100 Chieti, Italy; ^4^Department of Neuroscience, Imaging and Clinical Sciences, G. D’Annunzio University of Chieti-Pescara, 66100 Chieti, Italy

**Keywords:** atrial, amyloidosis, diagnosis, management, echocardiography

## Abstract

Amyloidosis is a systemic disease caused by low molecular weight protein accumulation in the extracellular space, which can lead to different degrees of damage, depending of the organ or tissue involved. The condition is defined cardiac amyloidosis (CA) when heart is affected, and it is associated with an unfavorable outcome. Different types of CA have been recognized, the most common (98%) are those associated with deposition of light chain (AL-CA), and the form secondary to transthyretin deposit. The latter can be classified into two types, a wild type (transthyretin amyloidosis wild type (ATTRwt)-CA), which mainly affects older adults, and the hereditary or variant type (ATTRh-CA or ATTRv-CA), which instead affects more often young people and is associated with genetic alterations. The atrial involvement can be isolated or linked to CA with a nonspecific clinical presentation represented by new onset atrial fibrillation (AF), diastolic dysfunction and heart failure with preserved ejection fraction, or thromboembolism and stroke. Untreated patients have a median survival rate of 9 years for AL-CA and 7 years for ATTR-CA. By contrast, AL-CA and ATTR-CA treated patients have a median survival rate of 24 and 10 years, respectively. Atrial involvement in CA is a common but poor studied event, and alterations of performance can anticipate the anatomical damage. Recently, numerous advances have been made in the diagnostic field with improvements in the available techniques. An early diagnosis therefore allows a more effective therapeutic strategy with a positive impact on prognosis and mortality rate. A multimodality approach to the diagnosis of atrial involvement from CA is therefore recommended, and standard echocardiography, advanced Doppler-echocardiography (DE) and cardiac magnetic resonance (CMR) can be useful to detect early signs of CA and to estabilish an appropriate treatment.

## 1. Introduction

Amyloidosis is a systemic disease caused by low molecular weight protein accumulation in the extracellular space, which can lead to different degrees of damage, depending on the organ or tissue involved. The condition is defined cardiac amyloidosis (CA) when heart is affected, and it is associated with an unfavorable outcome [1].

The recent improvement of diagnostic techniques has increased the clinical awareness and detection of this uncommon condition [1].

Different types of CA have been recognized, the most common (98%) are those associated with deposition of light chain (AL-CA), and the form secondary to a transthyretin deposit [2]. The latter can be classified into two types, a wild type (transthyretin amyloidosis wild type (ATTRwt)-CA), which mainly affect older adults, and the hereditary or variant type (ATTRh-CA or ATTRv-CA), which instead affects more often young people and is associated with genetic alterations [3].

There is scant information about the real prevalence of CA due to the small number of epidemiological studies, the different prevalence among the various types and the understimation of heart involvement in the clinical and imaging studies conducted so far (especially ATTRwt-CA). Despite these limits, ATTRwt-CA has a prevalence between 5.5% and 16.0% of older subjects (>80 years), ATTRv-CA is rarer and prevalence depends on a specific gene mutation; instead, AL-CA ranges from 15.5 to 40.5 cases per million in the United States (US) [4, 5, 6, 7, 8]. Clinical features are often not specific and can be misdiagnosed with those of other disease, such as aortic stenosis or heart failure with preserved ejection fraction (HF-PEF).

A recent European Society of Cardiology (ESC) position paper defines CA with the presence of left ventricular (LV) thickness ≥12 mm, with one or more of the following: autonomic dysfunction, peripheral polyneuropathy, hypotension or normotension if previously hypertensive, bilateral carpal tunnel syndrome, rupture of the biceps tendon, skin bruising and proteinuria, decreased QRS voltage to mass ratio, pseudo Q waves, AV conduction disease, late gadolinium enhancement (LGE) on cardiac magnetic resonance (CMR) or reduced longitudinal strain with apical sparing on echocardiography [2].

The atrial involvement can be isolated or linked to CA with a non specific clinical presentation represented by new onset atrial fibrillation (AF), ventricular diastolic dysfunction and HF-PEF, or thromboembolism and stroke [7].

Untreated patients with CA and atrial involvement from CA have a median survival rate of 9 years for AL-CA and 7 years for ATTR-CA. By contrast, AL-CA and ATTR-CA treated patients, have a median survival rate of 24 and 10 years, respectively [9].

Actually, complete epidemiological aspects of atrial involvement by CA are lacking, and at the moment only indirect data on the basis of retrospective studies are available. In a study performed by Bandera *et al*. [5] on 906 patients with ATTR-CA and subjected to speckle tracking echocardiography (STE), authors observed an impairment of all three phases of atrial function (reservoir, conduit and pump) with a total infiltration of atrial walls and absence of contraction up to 22% of subjects (n = 199 patients). Specifically, there was a reduction of reservoir, conduit and contraction function of 8.86% (5.94%–12.97%), 6.5% (4.53%–9.28%) and 4.0% (2.29%–6.56%), respectively [5].

In the recent years many authors focused on the role of making an appropriate pathway for an early identification of CA, with the aim of improving the prognosis and the survival [1, 6, 7, 8]: for example, in a retrospective study conducted by Brons *et al*. [6] on a total of 113 patients with CA, authors observed a greater number of diagnosed CA after implementing the diagnostic pathway (2019–2020 T2 vs. 2007–2018 T1). In the T2 period, number of CA diagnoses was 57 vs. 56 of the T1 period; this improvement was mainly due to a better attention to unexplained HF-PEF and to right ventricular (RV) hypertrophy (22% in T1 vs. 38% in T2 and 9% in T1 vs. 36% in T2, respectively). Moreover, this better clinical awarness led to a significant reduction of the diagnostic delay (14 vs. 8 months, *p* < 0.01 for T1 e T2, respectively), with less severe disease at diagnosis (New York Heart Association or NYHA Class III in 45% vs. 23%, *p* = 0.03 for T1 and T2, respectively), and minor CA stage (MAYO/Gillmore Stage III/IV; 61% vs. 33%, *p* < 0.01 for T1 and T2, respectively) [6].

Another retrospective study conducted by Tini *et al*. [7] on 1281 ATTRwt-CA patients from 17 Italian referral centres, authors noted that the diagnostic pathway that led to diagnosis was HF in 51% of patients (n = 651), incidental imaging in 23% (n = 300), incidental clinical in 19% (n = 236), HCM in 7% (n = 94); In opposite to other pathways, HF subjects were older (79 ± 7 years, hazard ratio (HR) 1.1, 95% confidence interval (CI) 1.1–1.2, *p* < 0.0001), had advanced III–IV NYHA class (HR 2.8, 95% CI 2.2–3.5, *p* < 0.0001) or chronic kidney disease (CKD) (HR 1.7, 95% CI 1.4–2.2, *p* < 0.0001) and showed worse survival and more comorbidities (especially AF, diabetes and chronic obstructive pulmonary disease or COPD) [7].

However information about the role of atrial imaging in patient with suspected CA and a possible ‘atrial pathway’ are lacking: a particular focus and attention to atria during diagnostic pathway in subjects with suspected CA and a multimodality approach to the diagnosis of atrial involvement from CA is therefore highly recommended.

Thus, the aim of this narrative review is to analyze and focus the use of standard echocardiography, advanced Doppler-echocardiography (DE) and CMR on the atria with the purpose to detect early signs of CA, to estabilish an appropriate treatment and to improve prognosis and survival [1].

## 2. Matherial and Methods

A search on PubMed/MEDLINE database was performed using the following keywords: ‘amyloidosis’ and/or ‘cardiac amyloidosis’, and/or ‘atrial amyloidosis’ or ‘isolated atrial amyloidosis’, and ‘echocardiography’ or ‘speckle tracking echocardiography’ and ‘cardiac magnetic resonance’, with a total of 490 articles. Two authors screened the articles (MT and AS). Only english language papers were included. A selection of studies was not performed due to the narrative nature of the review.

## 3. Patophysiology and Clinical Features

Amyloidogenesis consists of a dysregulation of the balance of formation and degradation of amyloid fibrils, with consequent accumulation of misfolded proteins that the organism is unable to remove. The deposits lead to the disruption of myocyte morphology and to architecture alterations, nodules and fibrosis with reduced vascularity [9, 10]. These alterations cause a reduction of atrial elasticity, contractility and emptying, and an increase of filling pressures. Furthermore, the abnormal ventricular tissue is less soft and contractile than normal, resulting in an increase of diastolic pressures and ventricular diastolic dysfunction.

The atrial alterations can be diffuse, focal or multifocal and can predispose to impairment of intramyocardial electrical conduction, resulting in re-entry and/or supraventricular arrhythmias, especially AF [11]. The latter can be secondary to atrial dilatation due to infiltration and thickening of the mitral valve [2, 9, 10].

These alterations predispose, even in sinus rhythm (SR) patients, to blood stasis and thrombosis with an increased risk of thromboembolic events such as stroke (Table 1).

**Table 1.** **General features of atrial amyloidosis**.

Anatomy	Function
Increased and thickened atrial walls	Restrictive pattern
increased and heterogenous echogenicity (“Speckling” or “sparkling” aspect)	- dilated atrial
	- small ventricles
	- diastolic impairment
	Alteration of all three phases of atrial function
	- reservoir
	- conduit
	- pump
Valve and tissue infiltrations	Variable regurgitation and sclerosis
Amyloid deposits, fibrosis and electrical isolation	Arrhythmias and AF
ANP overproduction	Blocks of various degrees
- Direct toxic effect by amyloid on endocardial tissue	Intracardiac thrombosis
- Activation of coagulation cascade	Thromboembolic events
- Accumulation of coagulation proteins (especially in AL-CA)	- stroke, TIA
- ‘Atrial standstill’	- Peripheral embolism
- Stasis due to bundle blocks	
Pericardial effusion	

ANP, atrial natriuretic peptide; AF, atrial fibrillation; AL-CA, light-chain cardiac amyloidosis; TIA, transient ischemic attack.

Thromboembolism is favored not only in patients with arrhythmias, but also in SR patients because atrial contraction is underperforming, a phenomenon called ‘atrial standstill’ which is relative to an electromechanical dissociation. Moreover, stasis may be favored also by Bachmann’s bundle envolvement with block of various degrees. In addition, the coagulation cascade may also be activated in SR subjects due to endothelial dysfunction secondary to fibrillar infiltration [10, 11, 12].

All of these factors can increase the risk of systemic embolic complications, such as transient ischemic attack (TIA) or stroke [13, 14, 15, 16].

### 3.1 Atrial Myopathy

Deposition of amyloid proteins in the extracellular space of the atria determines a progressive stiffening of the myocardial wall, resulting in structural fibrosis, loss of compliance and contractile function [1, 2, 3, 14].

The left atrium (LA) has three functional phases in healthy subjects, called ‘reservoir’, ‘conduit’ and ‘pump’ or ‘contraction’ phase, each determining the 50%, 30% and 20% of ventricular filling, respectively [1, 2, 3, 15].

Specifically, during the ‘reservoir’ phase the LA represents a ‘storage’ unit for energy when the LV is on isovolumetric contraction, ejection, and relaxation phase at mitral valve closed.

Conversely, the second or ‘conduit’ phase starts with the opening of atrioventricular (AV) valves at the beginning of the diastole and represents a specific ‘blood pathway’ from the pulmonary veins to the LV [14, 15].

Finally, the last phase, called ‘contraction’ or ‘pump’, permits, with the systolic ejection of the atrial myocardium, a further filling of the ventricular chamber at the end of the diastole.

All the three functional phases are impaired by amyloid deposits, especially the ‘reservoir’ one, thus resulting in decline of compliance and of systolic performance. It has also been hypothesized that ATTR-CA subjects may be more vulnerable to this process [14].

Furthermore, the atria involvement may represent a direct consequence of the high filling pressures secondary to LV diastolic dysfunction, or may occur simultaneously and separated from this, probably due to the direct deposition of amyloid in the extracellular space of atrial walls; this phenomenon, called ‘atrial myopathy’, leads to important consequences, such as the electrical isolation and the onset and manteinance of arrhythmias and AF, or to arterial thromboembolic events (AEs) such as stroke, TIA or peripheral vascular events [1, 14]. Moreover, some authors highlighted that the loss of atrial contraction can lead to rehospitalization, poor prognosis and higher mortality [14], therefore an early diagnosis by advanced ultrasound techniques of atrial dysfunction during the first phases of CA, in particular before the onset of ventricular infiltration, may be crucial to improve the prognosis of these patients [14, 15].

### 3.2 Thromboembolism and Acute Cerebrovascular Events

#### Intracardiac Thrombosis

It has been hypothesized that intracardiac thrombosis may have a multifactorial origin, other than blood stasis due to supraventricular arrhythmias. Amyloid fibrils can damage directly and have a toxic effect on endocardial tissue, by activating the platelet coagulation cascade and leading to intravascular thrombosis. Moreover, the nephrotic syndrome in AL-CA patients can lead to accumulation of coagulation proteins. As above discussed, an increased atrial stifness (‘atrial standstill’) or Bachmann’s bundle infiltration by amiloid fibrills can contribute to atrial thrombosis (Table 1) [7, 13, 14].

A comparative study conducted by Feng D *et al*. [16] on 116 total subjects with CA and undergoing autopsy, 38 of these (33%) had intracardiac thrombi, if compared to non CA group control. Non AL-CA group (n = 61) was oldest and had more AF; despite this, AL-CA group (n = 55) had more intracardiac thrombi (51% vs. 16%, *p* < 0.001, respectively) and fatal embolic events (26% and 8%, respectively with a *p* < 0.03); in addition, co-existence of AF and AL was more prone to develop thromboembolism with an odds ratio of 55.0 (95% CI 8.1–1131.4). With an odds ratio of 8.4 (95% CI 1.8–51.2) and 12.2 (95% CI 2.7–72.7) AL-CA group and LV diastolic dysfunction were independentely linked to embolic events, respectively [16].

An italian multicentric observational study conducted by Cappelli *et al*. [13] enrolled four-hundred-six subjects with CA (199 ATTRwt-CA, 73 ATTRm-CA and 134 AL-CA). Thirty-one of 406 patients (7.6%) had AEs and 10/31 of these (32%) were in SR. Twenty-nine patients had cerebrovascular events (21 ischemic strokes and 8 transient ischemic attacks) while 2 subjects had peripheral embolic events (1 femoral and 1 mesenteric). The most common CA subtype related to AEs was ATTRwt-CA (16 patients), followed by AL-CA (9 patients) and by ATTRv-CA (6 subjects). Moreover, there were thrombotic events in 14/185 patients (7.6%) despite optimal anticoagulation therapy and the only predictor of events in SR patients was a CHA2DS2-VASC score ≥3 (HR 2.84, 95% CI 1.02–7.92 in overall population; HR 10.13, 95% CI 1.12–91.19 in SR patients), thus suggesting the relevance of the CHA2DS2-VASC score in risk stratification for AEs of SR patients [13].

In a recent multicenter prospective study, Martinez-Naharro *et al*. [17]. evaluated 324 patients with CA (166 with ATTR-CA and 155 with AL-CA, 2 with apolipoprotein A-I, and 1 with apolipoprotein A-IV) and they found a prevalence of intracardiac thrombi of 7.2% (95% CI: 3.3%–11.2%), 5.2% (95% CI: 1.6%–8.7%) and 6.2% (95% CI: 3.5%–8.8%) in ATTR-CA group, AL-CA group and in overall population, respectively (*p* = 0.45).

The most common arrhythmia was AF (especially in patients with ATTR-CA vs. AL-CA: prevalence of 46.4% vs. 14.2%, respectively; *p* < 0.001) while atrial flutter had a prevalence only of 1.5%.

Moreover, ATTR-CA patients and AF had a prevalence of intracardiac thrombi of 14.3%, while AL-CA and AF of 9.1% (*p* = 0.52). All of these patients (intracardiac thrombi and AF) alread received anticoagulants (54% direct oral anticoagulants and 46% warfarin). In opposite patients with intracardiac thrombi and SR had a prevalence of 4.5% in AL-CA, and 1.1% in ATTR-CA (*p* = 0.11).

Thrombi were predominantly localized in the left atrial appendage (LAA) (90%), while only 6 patients had thrombi in other sites (30%). Severe biventricular systolic dysfunction (stroke volume *p* < 0.01; ejection fraction *p* < 0.05; mitral annular plane systolic excursion and tricuspid annular plane systolic excursion *p* < 0.01; and global longitudinal strain *p* < 0.01) was strickly correlated with a high risk of intracardiac thrombi [17]. In view of the high risk of thromboembolic events of atrial amyloidosis, the ESC, the American Heart Association (AHA), and the Canadian Cardiovascular Society/Canadian Heart Failure Society support the use of anticoagulants also in SR patients or in all patients with atrial and cardiac amyloidosis and concomitant arrhythmia, regardless CHA2DS2-VASc score. Ongoing studies are evaluating the best therapeutic choice (warfarin or non-vitamin K antagonists) [2, 18, 19].

### 3.3 Arrhythmias

Atrial involvement by CA represents a vulnerable substrate for the formation and maintenance of arrhythmias; amyloid deposits and fibrosis cause an electrical isolation among the myocytes and the onset of supraventricular arrhythmias (Table 1) [1, 2, 3, 4].

An impaired ventricular diastolic function and the increase of filling pressure lead to atria enlargement with further myocardial damage [11].

An altered diastolic relaxation of the ventricular cavity can be associated with an increase of atrial stretch and with the overproduction of atrial natriuretic peptide (ANP). ANP oligomers are therefore deposited between the myocytes and cause electrical isolation, impulse fragmentation and arrhythmias in a vicious circle [1, 10].

AF is very common in patients with CA: in a retrospective study conducted by Sanchis *et al*. [20], on 238 subjects [123 (52%) with ATTR-CA, 115 (48%) with AL-CA], 104/238 patients (44%) had history of AF; 42/104 patients (40%) had non permanent AF, and 62/104 patients (60%) had permanent AF. Fourty-eight patients had an episode of AF during the follow-up. Specifically, the most common CA subtype linked to AF was ATTRwt-CA (71%), while only 26% with AL-CA and 19% with ATTRv-CA had an episode of AF [20].

Another retrospective study conducted among 133 patients with CA (53% with AL-CA, 41% with wtATTR-CA, 6% with ATTRh-CA), confirmed that the most common subtype linked to AF was ATTRwt-CA (80%) vs. AL-CA (28%) and ATTRh-CA (13%) with a *p* < 0.001 and a 10-fold higher risk of AF. In opposite to CA patients with SR, those with AF were more frequently older (74 vs. 69 years, *p* < 0.001), male (80%) and had usually a NYHA symptom class ≥III (66%, *p* = 0.02). Moreover, patients with AF had more often comorbidities and severe symptoms. Curiosly, a direct correlation was noted between the prevalence of AF and advanced stages of ATTR-CA (47% vs. 74% vs. 94%, *p* < 0.001, for stages I, II, & III, respectively), while an inverse one with advances stages of AL-CA (0% vs. 40% vs. 31% vs. 18%, *p* < 0.001, for stages I, II, IIIa, & IIIb, respectively) [21].

Donnellan *et al*. [22] analyzed retrospectively 382 ATTR-CA patients in the period between 2004 and 2008 and 265 of these had AF, especially in an advanced stage of disease (69%). Elderly subjects, patients with higher stages of ATTR-CA, and higher left atrial volume index were all predisposing factors for AF onset and development. Moreover, they observed that a rhythm control strategy was more effective in the early stages of the disease; pharmacological and electrical cardioversion were used in in 35% and 45% CA patients with AF, respectively, while 5% of these was subjected to ablation. Advanced stages of CA were associated with worse prognosis and increased mortality, while maintenance of SR and tafamidis use were linked to better survival [22].

Given the risk of intracardiac thrombosis even in patients already treated with anticoagulant therapy, some authors suggest to perform in these subjects a transesophageal echocardiography (TEE) before electrical cardioversion [23, 24, 25].

It has been proposed also that drugs having a negative inotropic or chronotropic effect should be avoided, or at least used with caution at the minimum therapeutic dosage as rate or rhythm control; conversely amiodarone remains to be the best drug for rhythm control [10, 26, 27].

### 3.4 Isolated Atrial Amyloidosis (IAA)

IAA is an uncommon condition where amyloidosis affects directly the atria without any sign of ventricle involvement. Several authors suggest that this could be related to an abnormal ANP accumulation especially in the hearts of older patients. In a study conducted by Röcken *et al*. [28] on 245 subjects undergoing open heart surgery, the study of atrial appendages showed that IAA can predispose to AF through the infiltration of atria and conduction system by amyloid deposits: indeed, after Congo red staining and immunohistochemistry, 40/245 (16.3%) patients had amyloid proteins, which were all immunoreactive for ANP. Persistent AF was found in 38/245 (15.5%) subjects. Furthermore, patients with IAA and AF were at higher risk of prolonged P wave as compared with those with SR [28].

Moreover, Yang *et al*. [29] suggested that preamyloid oligomers formed by natriuretic peptides may have cytotoxic consequences and a proarrhythmic activity, and all of these effects are more evident in older adults, due to the physiological heart accumulation of natriuretic peptides.

## 4. Echocardiographic Features

### 4.1 Transthoracic Echocardiography

A very common finding is the thickening of the myocardial wall (> or = 12 mm), secondary to a diffuse infiltration of the myocardium by amyloid proteins and fibrotic tissue. Another characteristic that is not sensitive (35%) is the granular pattern of the myocardium (‘speckling or sparkling’). Despite a low diagnostic accuracy, the specificity of this finding is relatively high (80%) [30]. The sparkling aspect is secondary to the increased and heterogenous echogenicity related to the deposited amyloid fibrils [31].

**Fig. 1.**
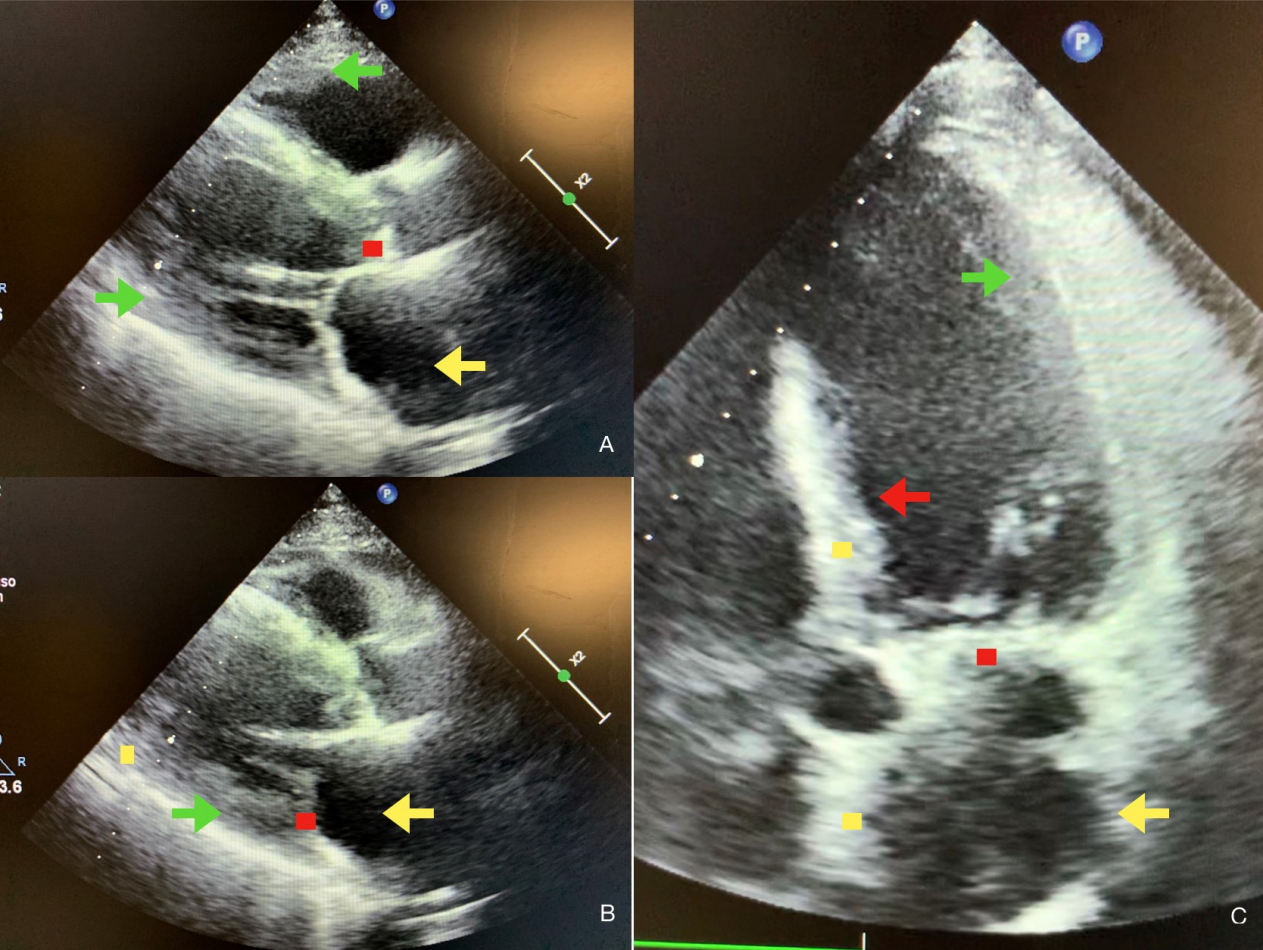
**Echocardiographic features of atrial amyloidosis**. Parasternal long-axis (A,B) and five chamber apical view (C) of cardiac amyloidosis (CA) and atrial amyloidosis characterized by the presence of concentric right and left ventricular thickness (green arrows in A, B, C), dilated and thickened atria (yellow arrows in A, B, C), by the thickening of the interventricular septum (red arrow in C), by sparkling spots in ventricular, atrial and septal walls (yellow dots in B, C) and by deposits in aortic and mitral valves (red dots in A, B, C).

Most common echo features are reported in Fig. 1 and in Tables 2,3.

**Table 2.** **Main echocardiographic, TDI and CMR findings of atrial amyloidosis**.

Echo	Strain echo imaging	TDI	CMR	Strain CMR imaging
Increased and thickened atrial walls	Reduced AS	Reduced a’ wave velocity <5 cm/sec	Increased and thickened atrial walls	Reduced reservoir, conduit, booster AS and reduced ASR
Increased and heterogenous echogenicity (“Speckling” or “sparkling” aspect)				
Valve infiltrations with variable regurgitation and sclerosis			Subendocardial LGE (“zebra-patten” like) with non-coronary distribution	
			Transmural LGE with non-coronary distribution	
			Increased T1 mapping and ECV values	
Restrictive configuration with dilated atrial, small ventricles, reduced ventricular cavity and diastolic impairment			Restrictive configuration with dilated atrial, small ventricles, reduced ventricular cavity	
Systolic dysfunction in later stages			Reduced emptying fraction	

TDI, tissue doppler imaging; CMR, cardiac resonance imaging; LGE, late gadolinium enhancement; ECV, extracellular volume; AS, atrial strain; ASR, atrial strain rate.

**Table 3.** **Atrial amyloidosis: red flags**.

Clinical scenario	ECG features	Echo features	Advanced echo features	CMR features
New onset AF	Decreased voltage to mass ratio	AV valve sclerosis or regurgitation	Reduced global and longitudinal strain	Restrictive pattern
HF-PEF	Atrial tachycardias, tachyarrhythmias (e.g., AF)	Restrictive pattern	Decreased TD velocities	Increased atrial walls thickness
Cerebrovascular events (e.g., stroke, TIA)		Increased atrial walls thickness		Atrial dilatation
Arterial peripheral events (e.g., femoral embolism)		Atrial dilatation		Diffuse LGE (subendocardial or transmural)
		Intracardiac thrombosis (e.g., LAA)		

ECG, electrocardiogram; AF, atrial fibrillation; HF-PEF, heart failure with preserved ejection fraction; TIA, transient ischemic attack; AV, atrioventricular; CMR, cardiac magnetic resonance; TD, tissue doppler; LGE, late gadolinium enhancement; LAA, left atrial appendage.

The interstitial accumulation of fibrotic tissue between the myocytes causes rigidity with reduction of ventricular compliance and various degrees of diastolic dysfunction [32, 33]. These abnormalities occur already in the early stages of the disease and tend to evolve gradually until the onset of a restrictive pattern [34] with an increased early diastolic peak (E) to atrial (A) ventricular filling velocities ratio >2 [35]. Only in the advanced stages of disease there can be a real loss of systolic function [36], a negative prognostic factor which is associated with a worse outcome [34].

Atrial dilatation is another unfavorable prognostic factor [37], and results from a reduced ventricular compliance, atrial wall and septum thickening from the myofibril deposits. Approximately, 60% of patients with amyloidosis have an increased atrial septum thickness [38]. A reduced atrial contractility is observed on echocardiography as a reduced or absent A wave [39].

Fibrillar deposition of the atrial walls leads to dilatation and thrombosis due to stasis, also found in patients with SR [40].

Heart valves are frequently thickened by amyloid deposits: 42% of patients with light chain amyloidosis had a thickening of >3 mm of the mitral valve at echocardiographic diagnosis. In a study, these patients were often elderly with an advanced NYHA class [37, 38, 39, 40, 41]. These alterations can be found also in the other forms of the disease, such as the transthyretin type [42] and have a negative prognostic significance [40, 43]. As discussed above, echocardiography is very useful to detect intracardiac thrombosis, which is located most often in the LAA [1, 13, 16, 17].

### 4.2 Strain Analysis with 2D Speckle Tracking Echocardiography

Some authors described alterations of atrial strain in all its functions (reservoir, conduit and pump) in 124 patients with ATTRwt-CA (27 patients), AL-CA (68 patients), ATTRm-CA (n = 29) and SR compared to twenty healty controls: all the functional phases of LA (longitudinal strain, early and late longitudinal strain rate or LSR, peak LSR) and LA active emptying fraction were altered, especially in the ATTRwt-CA subtype (*p* < 0.05). These alterations correlated with left ventricular deformation and strain: peak LA LS and late LSR were related with LV global LS (R = –0.60, *p* < 0.001) and with A wave at LV inflow (R = –0.69, *p* < 0.001), respectively [44].

Evaluation of atrium strain can be useful to differentiate CA from other disease with thickened myocardium from unclear cause, such as hypertensive heart disease. In a study conducted by Brand *et al*. [45], 54 subjects with thickened septal wall (17.8 ± 3.5 mm) were included: of these, CA was confirmed through biopsy in 35 patients (20 AL-CA, 8 ATTRm-CA, 6 ATTRwt-CA, 1 AA-CA) while left ventricular hypertrophy (LVH) in the remaining 19 patients. Specifically the reservoir, conduit and contraction strain of LA were reduced in CA group and were more accurate than apical sparing [45, 46].

A recent retrospective observational study performed from January 2019 to December 2022 by Monte *et al*. [15] highlighted how the function of the LA studied throught STE is significantly altered in CA patients if compared to healthy control group and in those with hypertrophic cardiomiopathy (HCM): they recruited a total of 100 patients (34 HCM, 33 ATTR-CA and 33 controls); the CA subgroup had impaired reservoir, conduit and contraction strain of the LA (median values of –9%, 6.7% and –3%, respectively) if compared to HCM patients and control group. LA volume index, LV mass index, E/e’, LV-global longitudinal strain correlated with the strain of LA and were strictly connected with dyspnea and AF [15].

### 4.3 Tissue Doppler Imaging

The recent position statement of the ESC Working Group on Myocardial and Pericardial Diseases confirmed the role of tissue doppler imaging (TDI) as non invasive echocardiographic criteria for diagnosis of CA, in particular the reduced tissue Doppler s’, e’, and a’ waves velocities (<5 cm/s) [2]. Koyama *et al*. [47] recruited 97 subjects with biopsy confirmed AL-CA and they divided them into 3 groups: those without cardiac infiltration (n = 36), those with heart infiltration and congestive heart failure (CHF) (n = 29) and those without CHF (n = 32). Main TDI finding are listed in Tables 2,3.

Tissue velocity, strain and strain rate imaging were measured by TDI: this technique showed significant differences among basal strain in all groups, with an early impairment of contractility before the onset of CHF in patients with cardiac infiltration by amyloid proteins. Specifically, transmitral flow peak a’ velocity, which expresses the end-diastolic ventricular relaxation secondary to atrial contraction, was generally reduced due to loss of atrial elasticity [47, 48].

Similar results were obtained in the study by Palka *et al*. [49], where 36 patients with biopsy proven CA were divided into two subgroups, those with non restrictive (n = 22) and those with restrictive (n = 14) LV filling pattern, and compared to a control group. All patients were subjected to TDI examinations that showed the reduction of mitral annulus velocities, incluse a’ wave velocity, the reduction of the mean myocardial velocities and of the myocardial velocity gradient, if compared to control group [49].

## 5. Cardiac Magnetic Resonance Imaging

### 5.1 Standard CMR

**Fig. 2.**
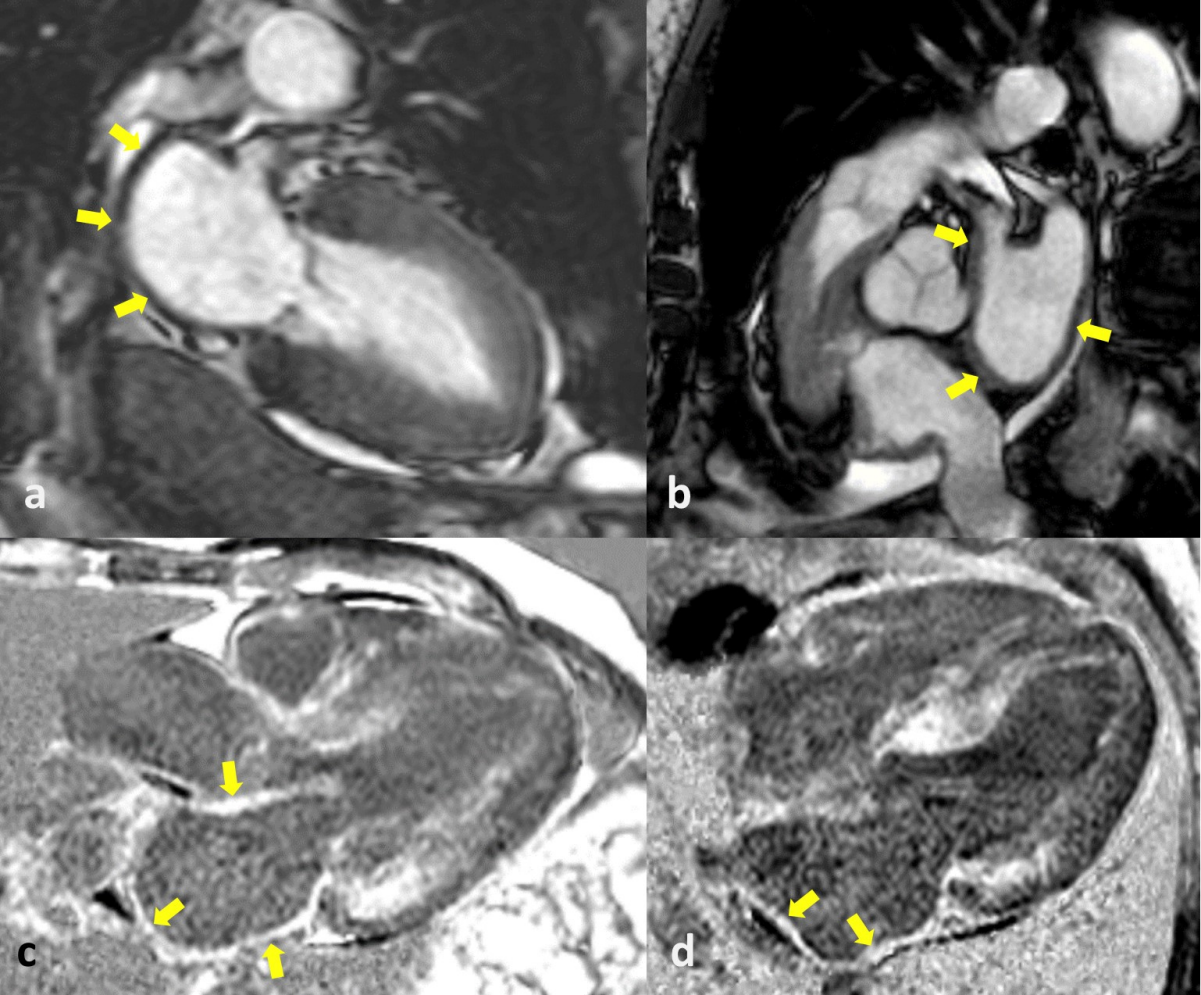
**Cardiac magnetic resonance (CMR) of cardiac amyloidosis (CA) and atrial amyloidosis**. The CMR images show a left atrium with thickened walls (yellow arrows in a,b) and a diffuse late gadolinium enhancement (LGE) of LA myocardium (yellow arrows in c,d). LA, left atrium.

As with the other cardiac structures, the atria chambers appear thickened, enhanced, and dilated on CMR [50, 51]. Most typical CMR features are reported in Fig. 2 and in Tables 2,3.

In a study performed by Di Bella *et al*. [52] on 28 patients (53 ± 12 years) with familial amyloid polyneouropathy (FAP), they observed that approximately half (14 patients) of them had LGE on CMR analysis if compared to 22 healthy control subjects (49 ± 11 years).

In a similar way, on twenty-two patients with biopsy-proven CA who underwent CMR, 17 of them (78%) showed a diffuse LGE of LA myocardium; the same authors postulate that LA LGE and atrial dysfunction may be correlated with the risk of thrombus formation [53].

Moreover, in another study which recruited 32 patients with ATTR-CA and 15 healthy controls, authors observed that left atrial dimensions were larger in patients with CA, and 9/10 patients with ATTR-CA vs. 0/8 HCM patients had LGE after CMR imaging (*p* < 0.001) [54].

LGE may be useful in the differential diagnosis with other cardiomyopathies, such as non-ischemic dilated cardiomyopathy (NIDC) or systemic hypertension (SH) [53]. Finally, CMR appears to have optimal resolution in detecting intracardiac thrombi [55].

### 5.2 Strain CMR 

CMR represents a valid and useful technique to evaluate the myocardial deformation; different methods have been developed to this function; one of these was the use of magnetic tags applied to the myocardium: tracking tag during cardiac cycle and visualization during CMR scans can provide useful information about the strain of the cardiac muscle, the strain rate and velocity of myocytes.

The same method can be used for atrial myocardium, diagnosing CA and monitoring treatment response.

Another technique is calculating the LA emptying functions, which is derived from the % reduction in LA volumes obtained by the validated biplane area-length method at the three diastolic phases (end-ventricular systole, pre-atrial contraction, and post-atrial contraction). These phases were characterized by aortic valve closure, second diastolic opening of mitral valve, and mitral valve closure [51, 52, 53].

CA patients recruited in the study by Kwong RY *et al*. [53] had a reduction of total LA emptying function (19 ± 14) if compared to SH and NIDC, respectively (40 ± 14 in SH subjects and 33 ± 20% in NIDC subjects, *p* = 0.0006) with a global reduction of active and total atrial emptying (r = –0.69, *p* = 0.001; r = –0.67, *p* = 0.01, respectively). These values were inversely proportional to LGE [53].

A study conducted on 44 patients with biopsy-proven CA, 19 with HCM and 24 healthy control subjects showed a reduction of reservoir left atrial strain (LAS), conduit LAS and booster LAS in CA and HCM subjects as compared to healthy control group (*p* < 0.001). Specifically, reservoir LAS and booster LAS were lower in CA patients in opposite to HCM group (*p* < 0.001) [56]. Similar results were obtained by Zhang *et al*. [57]: they included a total of 25 CA patients, 30 sex and age-matched hypertensive patients, 20 sane subjects, and studied LAS and LVS. Booster LAS, reservoir LAS, left atrial strain rate (LASR) were all impaired in CA if compared to healthy group (*p* < 0.001) [57].

Another retrospective study by Palmer *et al*. [58] on 54 patients (mean age 67 ± 11 years, 68.5% male) with CA (30 with AL-CA and 24 with ATTR-CA) compared to 15 age-marched control confirmed a marked reduction of the strain values of LA at CMR analysis vs. control group: in particular it was lower in ATTR-CA, with left atrial reservoir value of 7.4 (6.3–12.8) in ATTR-CA group if compared to 13.8 (6.90–24.8) in AL-CA group with *p* = 0.017; moreover booster strain value was 3.6 (2.6–5.5) in ATTR-CA patients and 5.2 (3.6–12.1) in AL-CA patients, with *p* = 0.039.

Moreover, LAS and left atrial emptying fraction (LAEF) were strickly correlated with amyloid burden in 43 AL-CA patients: in opposite to control group, AL-CA subjects had impaired LAEF and LAS, and larger LA volumes; this was more evident in patients at higher risk of disease (*n* = 27, high levels of troponine I and N-terminal pro-B-type natriuretic peptide (NT-proBNP)) [59].

In addition, some authors highlighted an impairment of both left and right atrial reservoir strain of CA vs. HCM subjects and control group [for RA; HCM group: 33.5 ± 16.3% vs. CA group: 10.6% (5.6; 19.9)], *p* < 0.001; [for LA; HCM group: 14.7 ± 7.1% vs. CA group: 7.0% (4.5; 11.1)], *p* < 0.001 [60].

Finally, in a study performed on 51 patients with proven CA, Benjamin *et al*. [61] also showed, in a median follow up of 4.9 months, a lower LA strain and higher LA volumes at CMR scans in opposite to 51 age-, gender-, and race-matched SR subjects and without cardiovascular disease (CVD).

## 6. Discussion

Atrial involvement by amyloid fibrils represents a particularly common event in patients with systemic and cardiac amyloidosis, causing walls thickening, dilatation of the atrial cavities, diastolic and systolic dysfunction. Furthermore, the fibrillar damage can also extend to the valve structures, thus amplifying the injury in a vicious circle. The infiltration of the conduction system generates an arrhythmogenic substrate, which is amplified by the reduction of contractility and atrial dilatation, thus resulting in thrombus formation and cardioembolism with cerebrovascular events.

Usually, the alterations of cardiac performance can anticipate the thickening and dilatation, therefore the doctor’s awareness appears fundamental for the purposes of an early diagnostic strategy and tailored therapy. As reccomended by Garcia-Pavia *et al*. [2], red flags represent helpful tools, and echocardiographic or CMR features of CA are well described. However, the role of atrial dysfunction in CA is not entirely involved for this purpose, and an accurate description and analysis may be further useful in this setting.

In addition, there is a lack of information about the role of atrial imaging in patients with suspected CA and a potential ‘atrial pathway’: various studies gave attention to right heart or to unexplained HF-PEF but a particular focus to atria during diagnostic pathway and a multimodality approach to the diagnosis of atrial involvement from CA is therefore highly recommended, so standard echocardiography, advanced DE and CMR aimed at studying the atria (even without a clear anatomical damage) can be very useful to detect early signs of the disease, to estabilish an appropriate treatment to improve prognosis and survival (Table 3).

Moreover, some grey areas in this field remain unexplored; for example, AF represents the most common arrhythmia in CA, but there are still few studies regarding other supraventricular arrhythmias. In addition, real prevalence and incidence of AF and other arrhythmias in CA are understimated, so occasional and systematic monitoring of the rhythm in SR patients with CA could be a valid preventive strategy.

As described, atrial amyloidosis represents a pro-arrhythmic substrate, that can easily lead to AF, but thrombotic events are possible in SR patients or despite anticoagulants; in this setting, anticoagulation, even in absence of arrhythmias, could be a sensible therapeutic approach, but randomized trials would be necessary. In addition, an elevated CHA2DS2-VASc score could find subjects at high risk of arterial thromboembolic events and may be an optimal tool in this setting.

Furthermore, early diagnosis is needed because treating the first stages of ATTR-CA can have greater success after electrical or pharmacological cardioversion, while advanced stages have poor prognosis, high mortality and poor response to cardioversion.

In this setting, non invasive diagnostic techniques, such as echocardiography and CMR, represent a valid and well studied strategy in detecting intracardiac and atrial thrombotic lesions, and they can be extended to SR patients with high thrombotic risk.

The article selection was limited to studies written in the English language. Both original and review articles were included in the present review.

## 7. Conclusions

Atrial involvement in CA is a common but poor studied event, and alterations of performance can anticipate the anatomical damage. Recently, numerous advances have been made in the diagnostic field with improvements in the available techniques. Therefore, an early diagnosis allows for a more effective therapeutic strategy with a positive impact on diagnostic delay, hospitalization, prognosis and mortality rate, but randomized studies are further needed.

Many authors highlighted the strict bond between CA and AF, especially for ATTRwt-CA, but information about the correlation among proven atrial amyloidosis and supraventricular arrhythmias are very low.

Cases of acute ischemic stroke in CA patients without a clear history of AF are described in literature, and greater attention should be paid to SR patients, who are also at greater risk of acute cerebrovascular events, especially those with higher CHA2DS2-VASc score.

In this setting, the use of non-invasive methods such as echocardiography and CMR can also represent a valid tool in the early diagnosis of intracardiac thrombosis.
